# To the Editors of the Medical and Physical Journal

**Published:** 1811-11

**Authors:** 


### *To the Editors of the Medical and Physical Journal.*

Gentlemen,

In a former volume you inserted a letter respecting the very meritorious "Society for the Relief of the Widows and Orphans of Medical Men in London and its vicinity;" this emboldens me to trouble you with a few lines respecting another Medical Society, which once enjoyed considerable estimation, but is now dwindled away to nothing. The Society to which I intend to call the attention of your readers is the "Lyceum Medicum Londinense," founded upwards of twenty years ago by the late *Dr. George Fordyce*, and *Mr. John Hunter*. This Society continued to flourish for many years; after the death of its founders the spirit of the Lyceum vanished, and several years have now elapsed since any regular meeting of the Members took place.

That a Medical Society of this kind should, after a time, fall into decay, is not much to be wondered at, however much it may be regretted; for it has happened to many Societies, much more celebrated than the Lyceum Medicum Londinense. It is not, therefore, to indulge in vain lamentations on this score that I now address you, but I am influenced from a consideration of compassion towards several unfortunate tradesmen, to whom this Society is indebted; and truly, Gentlemen, this is not a time when tradespeople can afford to lose their just demands. If, indeed, the Society had become insolvent, there would be nothing more to be said, the tradesmen must of necessity submit to the loss; but the Lyceum Medicum Londinense possesses money in the funds to a considerable amount, besides an extensive library, which is yearly becoming less valuable, thanks to the dust and to insects. Under these circumstances there is a paramount obligation attached to the surviving members of this Society, to see their creditors duly paid, for which purpose I venture to suggest that a general meeting of the Society be called, to order payment of the money justly due to their tradespeople, and afterwards to determine upon the proper means of disposing of the rest of their property. Perhaps the money could not be better employed than in encreasing the funds of the Society for the Relief of the Widows and Orphans above alluded to; nor the books be put to a better use than to be presented to some other Medical or scientific society; this, however, the members at large must decide upon.

I remain, Gentlemen,

Your obedient Servant,

A MEMBER of the Lyceum Medicum Londinense.

